# Removal of Porcine Endogenous Retroviruses in Decellularized Liver Bioscaffolds

**DOI:** 10.1111/xen.70097

**Published:** 2025-11-26

**Authors:** Elena V. A. van Hengel, Dubravka Drabek, Henk. P. Roest, Jorke Willemse, Lieve J. Reniers, Hera Stallmann, Jeroen de Jonge, Frank G. Grosveld, Luc J. W. van der Laan, Monique M. A. Verstegen

**Affiliations:** ^1^ Department of Surgery Erasmus MC Transplant Institute Rotterdam the Netherlands; ^2^ Department of Cell Biology Erasmus MC Rotterdam the Netherlands

**Keywords:** decellularization, extracellular matrix, liver bioengineering, porcine endogenous retroviruses, regenerative medicine, tissue engineering

## Abstract

Tissue engineering using decellularized liver scaffolds presents a promising approach in regenerative medicine, offering a potential alternative to donor organ transplantation. The use of human livers as a bioscaffold is restricted by their limited availability and quality. Porcine livers offer an alternative due to their anatomical and physiological similarities to human livers. However, applying porcine‐derived biomaterials in a clinical setting poses a risk of pathogen transmission, which is a noteworthy concern. Porcine endogenous retroviruses (PERVs), which are integrated into the genome of all pig breeds, are of particular concern, as subclasses PERV‐A and PERV‐B have shown to infect human cells in vitro. It is therefore essential to effectively remove all PERVs when manufacturing porcine scaffolds. In this study, we assessed the presence of PERV‐specific DNA, RNA, and protein in decellularized porcine livers. Our findings prove that genetic and protein PERV material was effectively removed from porcine livers during our decellularization procedure. This finding substantiates the potential of using decellularized scaffolds of porcine origin for clinical applications without risk of PERV transmission.

**Abbreviations**
ELISA: enzyme‐linked immunosorbent assay
*Env*
: envelope
FDA: Food and Drug Administration
*Gag*
: group‐specific antigen
GAPDH: glyceraldehyde 3‐phosphate dehydrogenase
PERV: porcine endogenous retrovirus
*Pol*
: polymerase
RT‐PCR: real‐time polymerase chain reaction

## 1 Introduction

Engineering functional liver tissue using decellularized livers offers a promising opportunity to address the gap between donor organ demand and supply in transplantation [1, 2]. During whole liver decellularization, the native extracellular matrix and organ architecture are preserved, creating a scaffold that supports cell repopulation to create a humanized graft. However, the use of human liver‐derived scaffolds is restricted by the shortage of available livers and quality variations [3]. Recent clinical studies of pig‐to‐human xenotransplantations have sparked renewed interest in the use of porcine biomaterials for regenerative medicine and tissue engineering [4, 5, 6]. Porcine livers are considered a suitable alternative source for liver‐derived scaffolds due to their anatomical and physiological similarities to human livers [7, 8, 9]. A major concern when using porcine scaffolds clinically is the risk of virus and pathogen transmission from pigs to human recipients. Extensive screening of donor animals for zoonotic viruses may help mitigate this risk, but it cannot eliminate porcine endogenous retroviruses (PERVs), which are integrated as proviral copies into the genome of all pigs [10, 11]. The potential presence of infectious PERVs in decellularized porcine liver scaffolds poses a critical challenge to their clinical application, due to the risk of zoonotic transmission and potential disease in human recipients [12, 13, 14, 15, 16, 17, 18]. A recent study on pig‐to‐human liver xenotransplantation suggests that targeted genetic modification and a dedicated pathogen‐free environment may have prevented PERV transmission, as no PERV was detected in the recipient [4]. PERV‐A and PERV‐B are found in all porcine breeds, although their viral expression varies by breed and tissue type [19, 20, 21]. PERV‐C can only infect porcine cells, whereas PERV‐A and PERV‐B have been shown to infect human cells in vitro [11, 19, 22, 23, 24, 25, 26, 27, 28]. PERV‐C is not present in all pig breeds, however, recombination with PERV‐A can give rise to PERV‐A/C recombinants. These somatic recombinants are infectious for human cells and are of concern in xenotransplantation research [26, 28]. A recent position paper by the International Xenotransplantation Association highlights the urgent need for regulatory frameworks and infection prevention strategies to enable the safe clinical application of pig‐derived materials and minimize the risk of PERV transmission [29]. Validated quantitative diagnostic assays are essential to detect PERVs in both source animals and xenograft recipients. A comprehensive strategy for PERV screening has been described using real time quantitative polymerase chain reaction (RT–qPCR) to detect viral RNA and antibodies to detect viral proteins using Western blot or enzyme‐linked immunosorbent assay (ELISA) targeting at least two different PERV proteins [30]. Current guidance by the United States Food and Drug Administration (FDA) for the use of xenotransplantation products, including cells, tissues, and organs, requires screening donor pigs for PERV DNA and RNA, and long‐term follow‐up of the recipient [31]. The recent FDA draft guidance outlines recommendations for the use of animal‐derived materials in tissue‐engineered medical products; however, it is of limited scope, addressing retroviral assessment solely through PCR [32].

**FIGURE 1:**
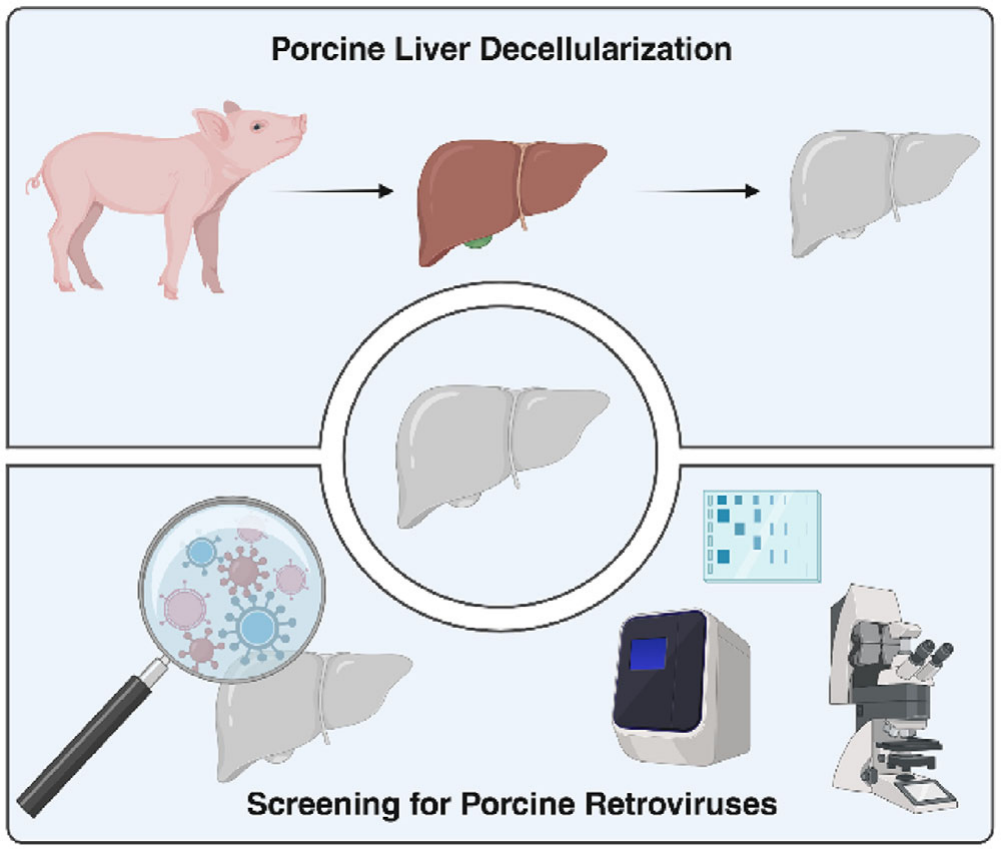
Schematic representation of porcine liver decellularization and screening for PERV. Porcine livers are obtained from the slaughterhouse and decellularized by perfusion using Triton X‐100 as described by Willemse et al. [35]. For future clinical applications in tissue engineering and regenerative medicine, the removal of PERV components during decellularization needs to be evaluated: the presence of PERV–*env* DNA is assessed using PCR, PERV–*pol* RNA is detected using RT–qPCR, and the presence of viral proteins is detected by immunofluorescence staining and Western blotting.

The genomic RNA of PERV encodes for three viral polyproteins: group‐specific antigen (*Gag*), polymerase (*Pol*), and envelope (*Env*) [25]. The PERV–*Gag* polyprotein is proteolytically cleaved into the individual structural proteins of the matrix, capsid, and nucleocapsid [25]. Assessing the presence of PERV genes and viral proteins is crucial to ensure the safe use of porcine decellularized liver scaffolds in tissue engineering and regenerative medicine (Figure 1) [12, 33, 34]. In this study, we selected three assays to determine the presence of PERV components in porcine livers before and after decellularization. First, PERV‐*env* copies in the porcine DNA were quantified, and PERV*‐pol* RNA was assessed using RT–qPCR. In addition, the presence of viral *Gag* structural proteins was determined using immunofluorescence and Western blotting, with antibodies targeting the matrix and capsid proteins. This study provides experimental evidence that whole‐liver decellularization effectively removes detectable PERV DNA, RNA, and proteins from porcine livers, thus supporting the feasibility of clinical translation.

## 2 Materials and Methods

Porcine livers (*N *= 6) were decellularized by continuous perfusion with 4% Triton X‐100 + 1% NH_3_, as previously described [35]. After DNA isolation and amplification, the presence of the PERV–*env* gene was determined using RT–qPCR and visualized using gel electrophoresis. In addition, the PCR product was further amplified using a nested PCR approach, and the amplicons were visualized on gel. Active PERV expression was determined in the scaffolds before and after decellularization, by the detection of viral PERV–*pol* RNA using RT–qPCR. The viral matrix domain protein of the *Gag* polyprotein (p15) and the major capsid protein (p27) were identified using immunofluorescence staining and Western Blotting. Full methods are described in Supplemental Materials and Methods.

## 3 Results and Discussion

**FIGURE 2:**
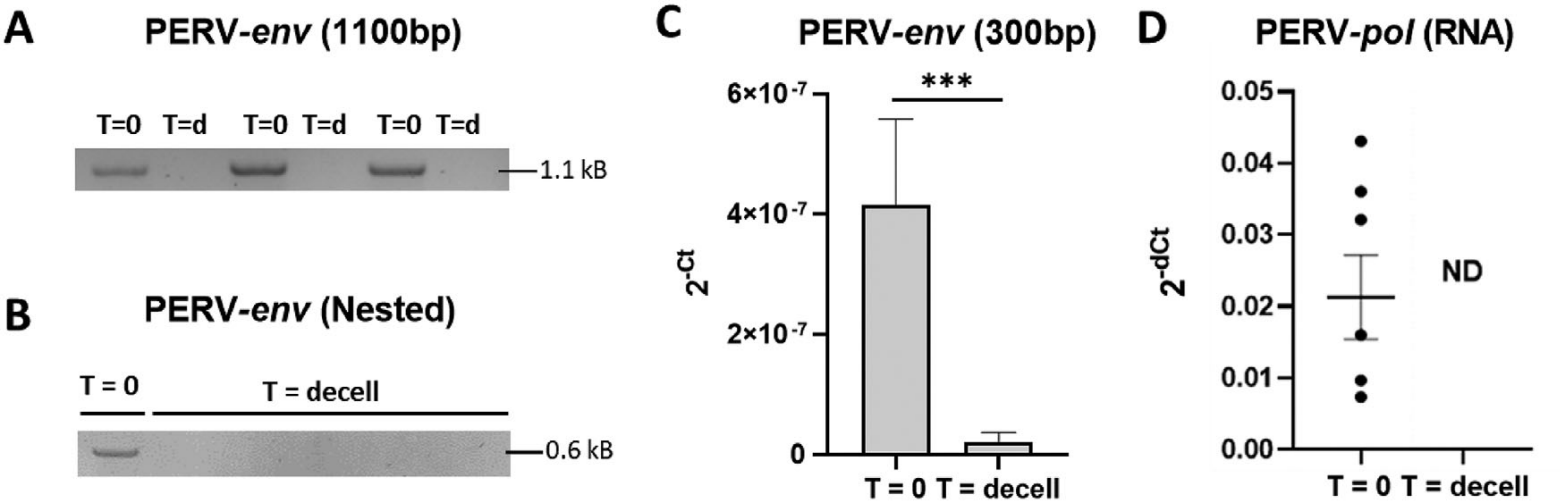
Decellularization effectively removes PERV genomic DNA and mRNA from porcine bioscaffolds. (A) PERV–*env* genomic DNA fragments of 1.1 kB amplicon size were clearly detected in pre‐decellularized liver tissue (*T* = 0), but were not detected after decellularization (*T* = d). (B) Nested PCR analyses of these samples remained negative for PERV–*env* DNA (amplicon size 0.6 kB). (C) Only very small DNA fragments (amplicon size 0.3 kB) of the PERV–*env* DNA were detected after decellularization using qPCR, however, levels were significantly lower. Relative expression is calculated as 2^−Ct^ as no normalization to a reference gene was performed. (D) Expression of the PERV–*pol* mRNA was not detected after decellularization by RT–qPCR analysis. ***p < 0.0001; ND, not detected.

After PCR amplification and gel electrophoresis, the 1.1 kB DNA amplicon was not detectable in decellularized scaffolds (Figure 2A). In addition, nested PCR, conducted with internally positioned primers, did not present any detectable amplification product on gel electrophoresis (Figure 2B). Using RT–qPCR, a significant decrease (*p *= 0.0005) in the 0.3 kB DNA amplicon of the PERV–*env* gene was determined after decellularization (Figure 2C). The RNA expression confirms that the decellularization protocol effectively removes the PERV–*pol* viral RNA (Figure 2D).

**FIGURE 3:**
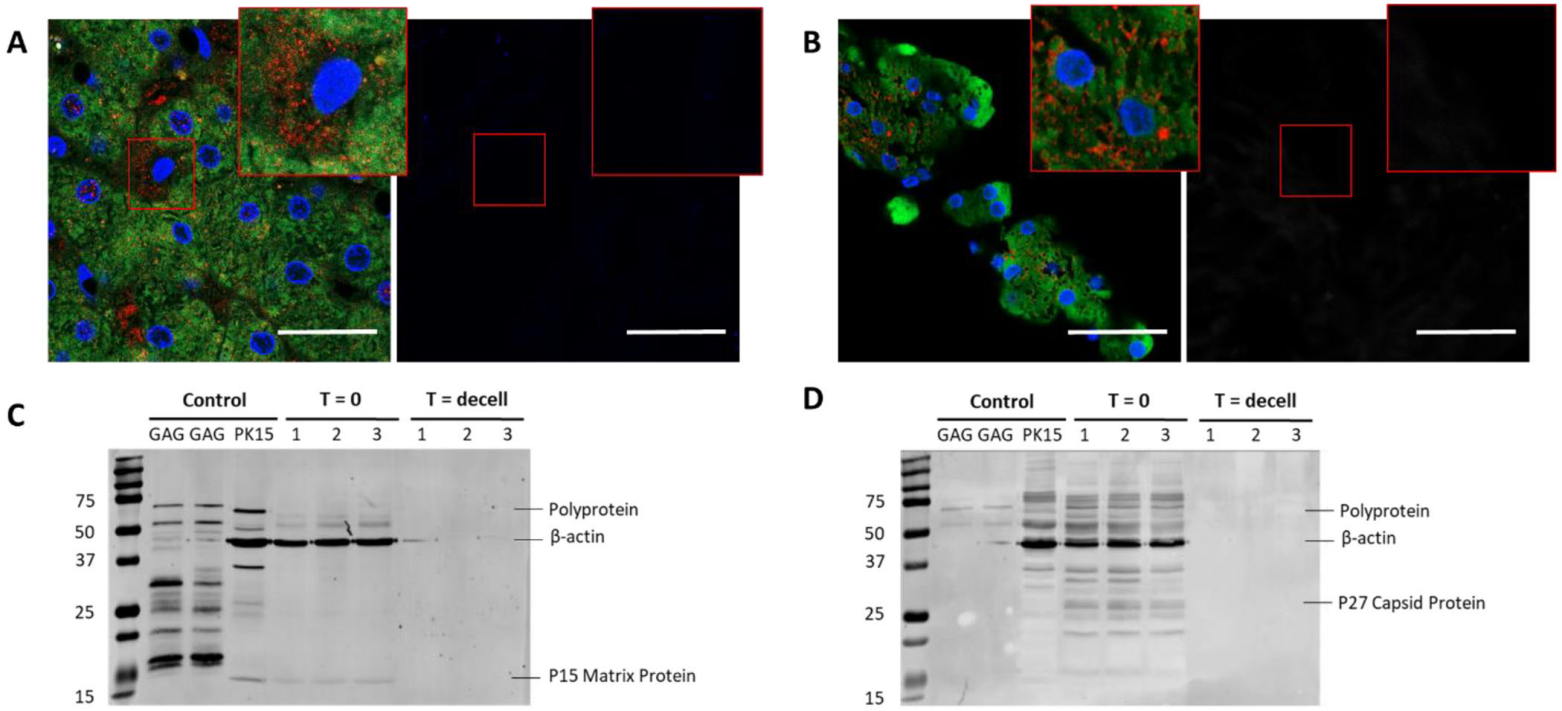
Decellularization effectively removes PERV viral proteins as determined by two specific antibodies. (A) Immunofluorescence staining of porcine liver tissue before (left) and after (right) decellularization, with cell F‐actin (green), nuclei (blue), and PERV–*Gag* p15 matrix protein (red) visible before decellularization. (B) Immunofluorescence staining before (left) and after (right) decellularization, with cell F‐actin (green), nuclei (blue), and PERV–*Gag* p27 major capsid protein (red). Both viral proteins are visible before decellularization but are eradicated after decellularization. Scale bars: 50 µm, *N* = 3. (C) Western blot analyses showing detectable p15 matrix protein and (D) p27 major capsid protein before decellularization but was absent after decellularization (*N* = 3). The presence of the PERV polyprotein and its cleaved products was detected with both antibodies in the *T* = 0 samples. The major capsid protein can appear at 27–30 kDa, depending on post‐translational modifications. Purified *Gag* samples and the PK15 cell line were included as positive controls for the antibodies. Detection of *β*‐actin (45 kDa) served as a control.

The PERV–*Gag* matrix protein (p15) and major capsid protein (p27) were determined by IF staining using two specific antibodies (Figure 3A,B), confirming their full removal as both viral proteins were visible before decellularization, but were not detected after decellularization. Additionally, the PERV polyprotein and its cleaved products were visualized using Western Blot (Figure 3C,D) and were not recognized after decellularization.

Although PERV pathology has been extensively studied, porcine‐to‐human xenotransplantation needs to be critically evaluated in (pre)clinical trials before being deemed safe [25, 33]. The importance of PERV detection for pig‐to‐human xenotransplantation was recently highlighted by the International Xenotransplantation Association [29, 30, 36]. Studies by others on porcine acellular vascular grafts and aortic valves did not show transmission of PERVs after implantation, reinforcing our data [13, 14]. Similarly, thorough evaluation of PERV genes and viral proteins remains essential for the safe application of pig bioscaffold‐based tissue engineering of humanized grafts and its clinical translation. Continuous monitoring of xenotransplanted organisms in experimental models is required before porcine liver‐derived scaffolds can be widely accepted for clinical use. We show that decellularization of porcine livers using our previously published protocol [35] is effective in completely removing viral PERV RNA, and the PERV–*Gag* viral proteins, suggesting that after decellularization, the transmission of PERVs is negligible, thus supporting the suitability of porcine livers as a source of liver‐derived scaffolds for tissue engineering.

## Author Contributions

E.V.A.H., L.J.W.L, J.J., and M.M.A.V. designed the study. J.J., M.M.A.V., and L.J.W.L. obtained funding. D.D. provided input on the experimental design and, together with F.G.G., produced and provided PERV antibodies and a PERV–*Gag* control sample and PK‐15 cells. J.W. and E.V.A.H. aided in the surgical procedures, procurement, and decellularization of the livers. E.V.A.H. performed immunofluorescence staining, confocal imaging, DNA isolation and analysis. L.J.R. isolated proteins and performed Western Blot. H.P.R. designed primers and H.P.R. and H.S. isolated RNA and performed and analyzed RT–qPCR. E.V.A.H. collected all data and drafted the figures. All authors have read, edited and approved the submitted version of the manuscript.

## Conflicts of Interest

Luc. J. W. van der Laan is a scientific advisor for HealthBanks Biomedical Taiwan, which is not related to the submitted work. All other authors have no conflicts of interest.

## Supporting information




**Table S1:** Primers used for genomic detection. **Table S2:** Primers used for quantitative expression analysis. **Table S3:** Antibodies for immunofluorescence staining. **Figure S1:** DNA fragments (amplicon 0.3 kB) of the PERV–*env* gene were detected after decellularization as visualized on gel electrophoresis (*N *= 3). **Figure S2:** (A) IF staining of PK15 cells with cell nuclei (blue), F‐actin (green), and Anti‐*Gag* Intracellular Protein (red). Scalebars: 50 µm. (B) Immunofluorescence staining of PK15 cells, with cell nuclei (blue), F‐actin (green), and Anti‐*Gag* Capsid Protein (red). Scalebars: 25 µm.

## Data Availability

The authors have nothing to report.
