# Supplementary material for: Removal of Porcine Endogenous Retroviruses in Decellularized Liver Bioscaffolds
Source: Xenotransplantation. 2025 Nov 26;32(6):e70097. doi: 10.1111/xen.70097 (PMC12679514; doi:10.1111/xen.70097)
Supplement: Supplementary file 1 — Table S1: Primers used for genomic detection. Table S2: Primers used for quantitative expression analysis. Table S3: Antibodies for immunofluorescence staining. Figure S1: DNA fragments (amplicon 0.3 kB) of the PERV–env gene were detected after decellularization as visualized on gel electrophoresis (N = 3). Figure S2: (A) IF staining of PK15 cells with cell nuclei (blue), F‐actin (green), and Anti‐Gag Intracellular Protein (red). Scalebars: 50 µm. (B) Immunofluorescence staining of PK15 cells, with cell nuclei (blue), F‐actin (green), and Anti‐Gag Capsid Protein (red). Scalebars: 25 µm. [file XEN-32-e70097-s001.docx]

**IN BRIEF**

**Removal of Porcine Endogenous Retroviruses in Decellularized Livers Bioscaffolds**

E.V.A. van Hengel^1^, D. Drabek^2^ & H.P. Roest^1^, J. Willemse^1^, L.J. Reniers^1^, H. Stallmann^1^, J. de Jonge^1^, F.G. Grosveld^2^, L.J.W. van der Laan^1^ & M.M.A. Verstegen^1^

^1^Erasmus MC Transplant Institute, Department of Surgery, Rotterdam, the Netherlands
^2^Erasmus MC, Department of Cell Biology, Rotterdam, the Netherlands

**Supplemental Materials and Methods**

**Methods decellularization**

Porcine livers (N=6) obtained from the slaughterhouse (Female German Landrace Pigs, 90-110 kg) were decellularized as described before. [34] In short, livers were cannulated via the hepatic artery and portal vein and connected to a peristaltic pump. The livers were pressure-controlled perfused with 20L dH_2_O, followed by continuous perfusion of 4% Triton X-100 + 1% NH_3_, and 5 cycles of reperfusion with 4% Triton X-100 + 1% NH_3_ for 120 minutes per cycle. Next, the livers were continuously perfused with 50L dH_2_O to remove remaining detergent. The livers were stored in 10L dH_2_O for 7-14 days, with water refreshment every 2 days. Lastly, livers were perfused with 5 mg/L DNase type I for 120 minutes. Decellularization was confirmed by quantification of DNA content (< 50 ng/mg tissue) and absence of cell nuclei after DAPI (4′,6-diamidino-2-phenylindole) and hematoxylin and eosin staining.

**Methods DNA expression**

DNA was isolated before and after decellularization using the QIAamp DNA Micro kit (Qiagen) according to manufacturer’s description and the yield was measured using a Nanodrop 2000. PCR amplification on 25 ng DNA per sample was performed following the GoTaq® DNA Polymerase Protocol (Promega) and thermal cycling protocol with 35 annealing cycles at 52 °C. Primers used are listed in Table S1. PCR products were separated on a 0.9% agarose gel using gel electrophoresis. In addition, a Nested PCR was performed using internally positioned primers (0.6 kB), and T=0 samples were diluted for a concentration curve to determine the lowest detectable amount of copy numbers. Statistical analysis was performed using Wilcoxon matched-pairs test.

**TABLE S1.** List of PCR primers

| **Primer** | **Amplicon length** | **Forward 5’ to 3’** | **Reverse 5’ to 3’** |
| --- | --- | --- | --- |
| PERV-*env* | 1.1 kB | AATCCCCTTAAGCTTCGCCTCCATCGC | AAGGTGTTGGTGGGATGGGGGA |
| PERV-*env* | 0.6 kB | TCCCCCATCCCACCAACACCTT | TCATGGAGTCTCTGATGGC |
| PERV-*env* | 0.3 kB | GCCATCAGAGACTCCATGA | AGTACCATGATCTGGACTGCACT |

**Methods RNA expression**

RNA was isolated before (N=6) and after (N=6) decellularization using the miRNeasy kit (Qiagen) according to manufacturer’s description and yield was measured using a Nanodrop 2000. cDNA was prepared using 5X PrimeScript Fast RT Master Mix on a 2730 Thermal cycler (Applied Biosystems). RT-qPCR was performed using the PowerTrack Masterix (Thermo fisher Scientific), on a StepOnePlus system (Applied Biosystems). Primers used are listed in Table S2.

**TABLE S2.** List of qPCR primers

| **Primer** | **Forward 5’ to 3’** | **Reverse 5’ to 3’** |
| --- | --- | --- |
| GAPDH | GATCGAGTTGGGGCTGTGACT | ACATGGCCTCCAAGGAGTAAGA |
| PERV*-pol* | CGACTGCCCCAAGGGTTCAA | TCTCTCCTGCAAATCTGGGCC |

**Methods Western Blot**

For Western Blot analysis before (N=3) and after (N=3) decellularization, 15 mg snap frozen tissue per sample was lysed in 900 µL ice cold RIPA buffer and homogenized with an electric homogenizer. Next, samples were placed on an orbital shaker to maintain constant agitation for 2 hours at 4°C. After centrifugation for 20 minutes at 12,000 rpm at 4°C, samples were gently removed and placed on ice. The supernatant was aspirated and placed in a fresh tube while kept on ice.

PK15 cells were grown in a cell culture flask and washed with ice-cold PBS before sample preparation. Ice cold RIPA buffer was added (1 mL per 10^7^ cells) and cells were scraped off the surface using a plastic cell scraper and transferred to a micro centrifuge tube. Samples were placed on an orbital shaker to maintain constant agitation for 30 min at 4°C, and afterwards centrifuged for 20 minutes at 12,000 rpm at 4°C. Samples were gently removed, the supernatant was aspirated and placed in a fresh tube kept on ice.

The sample and cell lysates and purified *gag* samples were diluted in 2x Laemmli loading buffer (1:1). Samples were heated for 5 minutes at 100°C. Experimental samples (N=6, wet weight 15 mg per sample), PK15 lysate (N=1, 20 µg protein), purified *gag* control samples (N=2, 100 ng) and marker (Precision Plus Protein™ Dual Color Standards, Bio-Rad cat#1610394) were loaded onto fresh prepared 12% SDS-PAGE gels and subsequently transferred onto Immobilon-PVDF membranes (Millipore) via electroblotting. The proteins in the membranes were blocked using Odyssey blocking buffer (cat. #927-70001, Licor-Biosciences) and incubated overnight at 4°C with primary antibodies A5 Anti-*gag* p15 Matrix Protein (mouse, 1:1000), 4G3 Anti-gag p27 Major Capsid Protein (mouse, 1:1000) and β-actin (1:5000; ab20272, Abcam). Both antibodies were engineered into full length Heavy chain only format with mouse IgG1 Fc. A5 llama single domain antibody against gag p15 was described in [11]. The 4G3 anti-*gag* p27 was obtained by screening a llama synthetic library from Unilever. After washing in PBS-0.05% Tween, the membranes were incubated with secondary antibody (Goat anti-mouse Alexa Fluor 555, 1:2500, cat. #A-21422) at room temperature for 1 hour. Gels were visualized on an Odyssey® Fc Imaging System.

**Methods immunofluorescence staining**

Biopsies from porcine livers (N=6) obtained from the slaughterhouse and decellularized porcine livers (N=3) where fixed in paraformaldehyde 4% and washed in PBS 1X. Formalin-fixed paraffin-embedded samples were sectioned (4 µm thick) according to standard procedures. After deparaffinization using Xylene, 100% ethanol, and 70% ethanol, antigen retrieval was performed by cooking the samples in Citric Acid buffer. PK15 cells were cultured on cover slides coated with 0.1% w/v% gelatin and used as a positive control. After washing with 0.025% Triton X-100 in PBS 1x, a PAP pen was used to surround samples before blocking using 10% goat serum.

The heavy chain antibodies against A5 Ilama Anti-*gag* (p15) and 4G3 Anti-*gag* (p27) were prepared as described in [11]. Primary antibodies were incubated overnight at 4˚ C and washed with 0.025% Triton X-100 in PBS 1x. Secondary antibody was incubated for 60 minutes at RT. Remaining antibody was washed away using PBS 1x, and Phalloidin was added for 20 minutes at RT. Using a droplet of DAPI Vectashield (VectorLabs) a cover glass was affirmed on top. Samples were imaged with a Zeiss LSM 900 fluorescence microscope (40x – 400x magnification).

**TABLE S3. Antibodies for immunofluorescence staining**

| **Primary Antibody** | **Origin** | **Concentration** |
| --- | --- | --- |
| Anti-g*ag* p15 (Matrix Protein) | Mouse | 1:100 |
| Anti-*gag* p27 (Major Capsid Protein) | Mouse | 1:100 |
|  |  |  |
| **Secondary Antibody** |  |  |
| Goat anti-mouse IgG | Alexa Fluor 555 | 1:100 |
| Phalloidin | Alexa Fluor 488 | 3:100 |

**Supplemental Results**


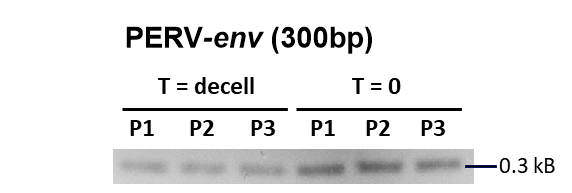


**FIGURE S1.** DNA fragments (amplicon 0.3 kB) of the PERV-*env* gene were detected after decellularization as visualized on gel electrophoresis (N=3).

**Control Immunofluorescence staining**

The porcine kidney cell line PK15 spontaneously releases PERV particles and was used as positive control for p15 structural matrix protein and p27 major capsid protein expression. [21]


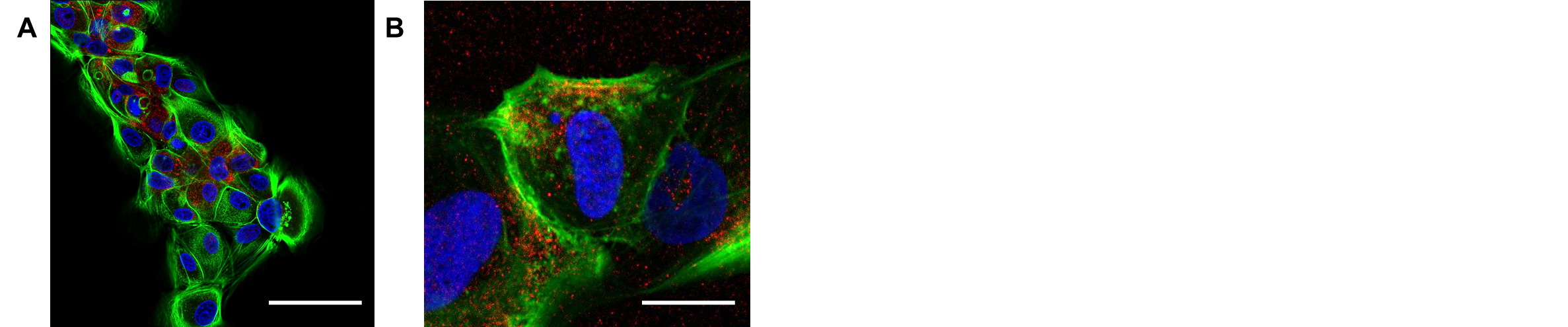


**FIGURE S2.** (A) IF staining of PK15 cells with cell nuclei (blue), F-actin (green), and Anti-*gag* Intracellular Protein (red). Scalebars: 50 μm. (B) Immunofluorescence staining of PK15 cells, with cell nuclei (blue), F-actin (green), and Anti-g*ag* Capsid Protein (red). Scalebars: 25 μm.
